# Attachment Manifestations in Daily Interpersonal Interactions

**DOI:** 10.1007/s42761-022-00117-6

**Published:** 2022-07-14

**Authors:** Aleksandra Kaurin, Paul A. Pilkonis, Aidan G. C. Wright

**Affiliations:** 1 Faculty of Health/School of Psychology and Psychiatry, Witten/Herdecke University, Alfred-Herrhausen-Straße 44, 58455 Witten, Germany; 2 Department of Psychiatry, University of Pittsburgh School of Medicine, Pittsburgh, PA USA; 3 Department of Psychology, University of Pittsburgh, Pittsburgh, PA USA

**Keywords:** Attachment, Ambulatory assessment, Multilevel structural equation modelling, Interpersonal theory, Working models

## Abstract

The predominant focus in attachment research on trait-like individual differences has overshadowed investigation of the ways in which working models of attachment represent dynamic, interpersonally responsive socio-affective systems. Intensive longitudinal designs extend previous work by evaluating to what extent attachment varies over social interactions and the functional processes that underlie its fluctuation. We examined momentary activation of attachment orientations in the stream of peoples’ daily lives and how those patterns were linked to interpersonal behavior. Based on an event-contingent, ambulatory 7-day assessment protocol (*N*=263; 3,971 interactions) operationalized using Contemporary Integrative Interpersonal Theory, we examined whether contextually activated working models accounted for patterns of interpersonal (anti-)complementarity. Our analyses revealed that the situational activation of working models varied as a function of interpersonal perceptions of warmth, which were linked to greater state security and lower levels of anxious or avoidant expectations. These reactivity patterns, in turn, accounted for interpersonal complementarity. Avoidant attachment was linked to diminished and secure attachment to enhanced expressions of warmth. The analyses were robust even when controlling for momentary positive and negative affect and closeness of the relationship. Attachment expectations wax and wane across daily social interactions, and such fluctuations are reflective primarily of a process in which perceptions of others’ warmth activate secure attachment expectations and lower insecure ones.

**Supplementary Information:**

The online version contains supplementary material available at 10.1007/s42761-022-00117-6.

Attachment orientations represent dynamic, socio-affective systems that are activated in actual or imagined interpersonal interactions and reflect an individual’s expectations of how others will perceive and behave toward them (Bowlby, 1969, 1980; *see also* Fraley & Brumbaugh, 2004). The contextually responsive quality of attachment expectations or internal working models (Mikulincer & Shaver, 2003), however, has not been assessed within the stream of meaningful everyday social interactions. Instead, research has conventionally operationalized attachment as an individual difference, presumed to generalize over interpersonal situations (Gillath et al., 2009). Thus, what cannot be ascertained from previous studies is how attachment expectations manifest in interpersonal interactions in peoples’ daily lives. Consequently, we know little about the functional role of *momentary* attachment manifestations for daily interpersonal processes. The current study sought to fill this gap. It provides a methodological articulation of attachment that aligns with concepts of working models as contextualized, interpersonally responsive dynamic systems via a 7-day event-contingent ambulatory assessment protocol.

Attachment theory provides an explanatory framework for understanding the ways in which people initiate and maintain social relationships (Bowlby, 1969; Collins & Allard, 2001). Traditionally, individual differences in attachment have been structured along two dimensions, anxiety and avoidance (Brennan et al., 1998; Crowell et al., 1999), with securely attached individuals presumably low in both. Conceptually, each of the two attachment dimensions is related to a distinct set of socio-affective processes reflecting beliefs about the availability, trustworthiness, and supportiveness of others along with characteristic patterns of interpersonal perceptions, behaviors, and affect-regulatory strategies (Brennan et al., 1998). In interpersonally threatening or stressful situations, anxious individuals tend to engage in hyperactivating strategies such as hypervigilance and pressured attempts at approach and attachment, whereas avoidant individuals tend to engage in deactivating strategies such as keeping others at a distance (Davila & Kashy, 2009; Mikulincer & Shaver, 2003). In times of distress, securely attached individuals tend to seek interpersonal comfort and support in more adaptive ways (Bowlby, 1969; Bretherton & Munholland, 2008).

Adult attachment is assumed to originate from early caregiving experiences and is often conceptualized in terms of stable individual differences (Gillath et al., 2009). However, contemporary theoretical approaches challenge this assumption, proposing instead that adult attachment styles are also shaped by recent interpersonal experiences in a continuously updating process throughout development (Fraley & Roisman, 2019). This perspective suggests that attachment is a dynamic system that not only influences relationships and interactions but is influenced by them as individuals go about their daily lives.

From the outset, working models of attachment have been defined as flexible relational schemas, likely to be displayed in heterogeneous ways across diverse everyday relational contexts (Bowlby, 1969). Such plasticity results from working models that represent an ensemble of both activating and deactivating behaviors, structured in ways that facilitate one’s preferred balance of attachment and autonomy (Mikulincer & Shaver, 2003). In the past, monthly or yearly fluctuations in attachment have been linked to declines in relationship satisfaction, increases in relationship distress (Girme et al., 2018), and personality pathology (Davila et al., 1997). People, however, hold a variety of working models to accommodate multiple attachment figures and can access contextually relevant guidance for different attachment needs that arise across time (Baldwin & Fehr, 1995; see also Doherty & Feeney, 2004; Fraley et al., 2011; Girme & Overall, 2021; Overall et al., 2003).

Investigating this systematic variation in ways that map onto the dynamic and interpersonal nature of attachment is important because what may appear to be discontinuity over time may not be reflective of general patterns of change per se but a situation-specific manifestation of working models (e.g., Baldwin et al., 1996; Fraley & Roisman﻿, 2019). In therapy, such situation-specific activation can be used to facilitate change (Levy et al., 2018; Tasca & Balfour, 2014) through the provision of context-specific interpersonal experiences (Mallinckrodt, 2010).

Some studies have experimentally examined such patterns of within-person fluctuations of attachment in the context of stressful interpersonal experiences. Having participants recall an occasion during which they felt unloved or disrespected by an attachment figure, as well as experimentally induced relational distress with a key attachment figure, has been associated with temporary decreases in attachment security (Bosmans, Bowles, et al., 2014; Bosmans, Van de Walle et al., 2014; Vandevivere et al., 2018; Verhees et al., 2020). Similarly, asking participants to imagine someone very supportive in their lives reduced the vividness and distress related to intrusive memories, especially so in participants characterized by less avoidant attachment (Bryant & Datta, 2019). Such results on the effects of attachment activation were extended by Schreiber et al. (2021), who examined associations between moment-to-moment attachment dynamics and patterns of physiological coregulation in an observational study. In their study, increases in state attachment avoidance were associated with a desynchronization of partners’ heart rates, whereas increases in state attachment anxiety were associated with synchronization during a laboratory discussion task.

Laboratory observation provides a valuable vantage point, but it tells us little about how attachment expectations influence interpersonal processes in daily life. Thus far, only a few studies have examined the variable impact of attachment expectations in people’s daily lives. Over the course of 8 weeks, Davila and Sargent (2003) observed that on days when people perceived significant attachment figures to be unavailable, they also tended to experience more attachment insecurity. Similarly, based on a shorter, 1-week protocol of daily assessments, Haak et al. (2017) found that on days when participants felt more accepted by their romantic partners, they reported lower levels of anxiety and avoidance. In addition, perceptions of acceptance predicted the next day’s attachment security, which was accounted for by lowered negative affect. Finally, an analysis of attachment states and relationship functioning collected twice a week for 4 weeks found that daily experiences of lacking emotional support, trust, closeness, affection, friendship, or companionship were associated with attachment anxiety, but not avoidance (Zhang, 2009).

Although it appears that attachment expectations fluctuate in systematic ways across everyday social experiences, previous work did not link the activation of working models to specific interpersonal interactions as they unfold in real-world contexts. To address this gap in the empirical literature, we applied Contemporary Integrative Interpersonal Theory (CIIT; Hopwood et al., 2021; Wright et al., 2020) as a translational framework to address some of the challenges described here and assess attachment processes as they unfold in the daily social lives of individuals. As an integrative model, CIIT encompasses the mental representations central to attachment theory such as the perceived availability or supportiveness of others (in terms of affiliation or dominance) and how that perception is tied to one’s own interpersonal behavioral response. More specifically, CIIT rests on the assumption that individual differences (such as those in attachment orientations) emerge from generalized patterns of mental construal (i.e., the way in which we perceive social cues) and from our responses to the proximal behavior of others (Kiesler, 1996; Wright et al., 2017).

Previous work (Leary, 1957; Wiggins, 1991) has established two independent dimensions—agency and communion—organizing interpersonal perceptions and behavior. Together they define the interpersonal circumplex, which encompasses attributes of agency and communion. Agency refers to tendencies to be in control vs. tendencies to be passive and seek guidance from others (i.e., dominance vs. submissiveness). Communion refers to tendencies to seek proximity to others vs. tendencies to be distant and socially unapproachable (i.e., affiliation vs. quarrelsomeness). The circumplex structure serves the purpose of identifying how different patterns of interpersonal behavior relate to each other (Zimmermann & Wright, 2017) and can be used to empirically distinguish behavioral signatures of mental representations such as those related to attachment states in terms of their prominent interpersonal themes (e.g., Kaurin, Beeney, Stepp et al.,^ 2020^; Sadikaj et al., 2011). One of the pillars of CIIT is the principle of *complementarity* (Wiggins, 1991; Wright et al., 2020). It contends that each interpersonal behavior has a counterpart characterized by a similar amount of communion and an opposite amount of agency. To illustrate, dominant behavior tends to elicit or invite submissive behavior, whereas warm behavior tends to elicit or invite warm behavior (Carson, 1969). Interactions in which opposite behavioral patterns emerge (e.g., perceived warmth elicits less of one’s own warmth; perceived dominance is met with one’s own dominance) are referred to as anti-complementary (Kiesler, 1983). Interactions marked by anti-complementarity are hypothesized to be experienced as aversive, whereas those characterized by at least a moderate amount of complementarity are generally perceived as pleasant (Carson, 1969). These principles can be elaborated and combined with key components of other psychological domains within interpersonal situations such as attachment fluctuations. They have also been used to describe characteristic interpersonal transaction cycles in the context of individual differences such as in personality (Fournier et al., 2009), psychopathology (Benjamin, 1996; Hopwood, 2018), or dispositional attachment orientations (Kaurin, Beeney, Stepp et al., 2020; Sadikaj et al., 2011). Thus, while examining dynamic within-person sequences of interpersonal experience, we used CIIT as a framework to operationalize patterns associated with situationally fluctuating attachment states.

## The Current Study

**Fig. 1:**
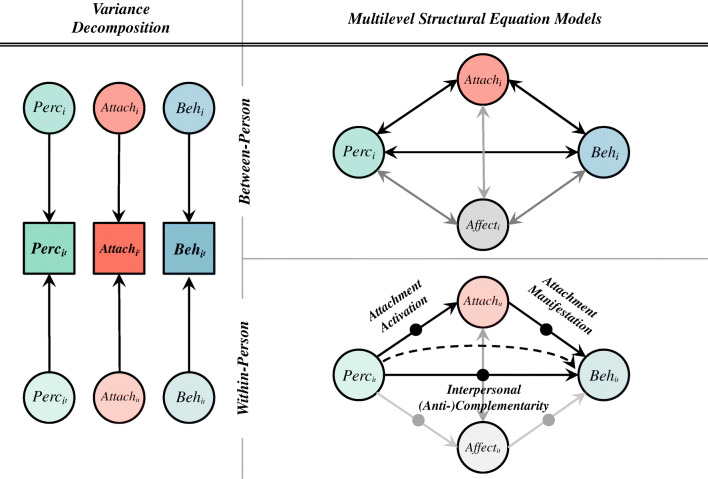
Schematic overview of estimated multilevel structural equation models. The panel on the left depicts the latent decomposition of observed momentary variables into between- (subscript *i*) and within-person (subscript *it*) variance. The right panel depicts the within-person (bottom) and between-person (top) portion of our focal model. Grey paths denote sensitivity analyses as presented in Tables 3, 4, and 5. Solid dots represent random slopes on within-person regression paths. *Perc*—perceived affiliation or dominance; *Beh—*own affiliative or dominant behavior; *Attach*—attachment. Parameters not reported in the tables (e.g., residual variances, covariates) are not depicted in diagrams, but respective Mplus code providing full models specification can be found online at: https://osf.io/3vy4m/

The purpose of this study was to provide a methodological articulation of attachment that aligns with a conceptualization of working models as dynamic systems of interpersonal expectations (Bowlby, 1969; Mikulincer & Shaver, 2003). To this end, we assessed the interpersonal context (as conceptualized in CIIT) that characterizes the variation of attachment experiences in everyday interpersonal interactions based on a 7-day event-contingent ecological momentary assessment (EMA) protocol. We used CIIT, because it provides a set of concepts to empirically test specific instantiations of mental representations in social interactions in daily life. Effects of individual differences in attachment orientations were distinguished from effects at the level of social interactions via multilevel structural equation modelling (MSEM). In line with CIIT, we evaluated whether and to what extent perceptual and behavioral features of interpersonal interactions were linked to attachment expectations in daily life. Consistent with CIIT’s focus, we sampled perceptions and behaviors using the dimensions of dominance and warmth. We then examined within-person models to test whether attachment states that varied across situations were linked to differential affective and interpersonal responses. More specifically, to provide insight into social situations characteristic of insecure attachment, we examined whether reactive changes in attachment accounted for *interpersonal (anti-)complementarity* via two critical pathways: The momentary elevation of state attachment in response to perceptions of one’s interaction partner (*attachment activation*) and the unfolding interpersonal behavioral response (*attachment manifestation*; see Figure 1).

We hypothesized that situational fluctuations of attachment would be associated with the perceived nature of the interaction (i.e., dominance, warmth) such that they would reflect characteristic patterns of interpersonal perceptions, behaviors, and regulatory strategies (Brennan et al., 1998). Our prediction is based on previous work proposing that patterns of interpersonal perceptions and behaviors vary as a function of attachment orientations (e.g., Fraley & Brumbaugh, 2004; Kaurin, Beeney, Stepp et al., 2020). It is also informed by work suggesting that in interpersonally stressful situations, anxious individuals tend to engage in pressured attempts at approach, avoidant individuals tend to keep others at a distance (Davila & Kashy, 2009; Mikulincer & Shaver, 2003), and securely attached individuals tend to seek interpersonal comfort and support in more adaptive ways (Bowlby, 1988; Bretherton & Munholland, 2008). Specifically, we hypothesized that when interactions were characterized by a strong availability and supportiveness of an interaction partner via perceived interpersonal warmth, higher security and lower anxiety and avoidance would be observed. Conversely, in interactions characterized by a lack of availability and supportiveness such as via perceived interpersonal dominance, lower security and higher anxiety and avoidance would be observed. Because of these relationships, we further hypothesized that avoidant attachment states would covary with dominant, interpersonally cold and distant behavior, whereas anxious attachment states would more likely be linked to submissiveness and proximity-seeking, as an indicator of characteristic hyperactivation strategies. Secure attachment states, in turn, would be characterized by warm and affiliative interpersonal behavior.

## Method

All study procedures were approved by the Institutional Review Boards of the University of Pittsburgh (IRB Protocol #: STUDY20090074). This study was not preregistered and all data have been made publicly available at the OSF and can be accessed at https://osf.io/3vy4m/. We report how we determined our sample size, all data exclusions, and all measures in the study.

### Sample

Undergraduate students (*N*=263, reporting 3,971 social interactions)^1^ were recruited online from introductory psychology courses at the University of Pittsburgh. Participants were ~57% female and ranged in age from 18 to 27, with the majority (87.3%) being 18 and 19 years old (*M*=18.81, *SD*=1.98). Most participants identified as White (66.53%, *n*=175), 17.87% as Asian (*n* =47), 7.22% as Black (*n* =19), and the remainder as other. Participants received course credit for completing the baseline questionnaires and EMA protocol. Full credit was awarded to individuals who completed an average of four or more surveys per day. This number is based on extensive prior use of these methods. It reflects the average amount that people have reported in earlier studies and matches a cutoff of ~70% of surveys completed used in related studies (e.g., Kaurin, Dombrovski, Hallquist 2020).

### Sample Size Justification

Two aspects guided our selection of the target sample size: First, our dataset was intended to serve as resource that can be examined to answer many questions expected to range in effect size. We anticipated that most analyses would be based on covariance matrices like those described in the present paper. Therefore, the choice of our target sample size was most strongly influenced by our appeal to derive stable effect estimates, in contrast to having the power to detect any specific effect size in the population. Recent work has suggested that correlation estimates consistent with the average effect in the literature on individual differences begin to stabilize when sample sizes approach *N*=250 (Schönbrodt & Perugini, 2013). Thus, for our most conservative tests, which would be between-person associations given the hierarchical structure of the data (interactions nested within persons), we sought a minimum sample size of *N*=250. The within-person portions of the models would be expected to far exceed this number given each participant reported on multiple interactions.

### Procedure

Participants viewed a video-assisted training presentation explaining the EMA procedures and instructions for downloading the *MetricWire* smartphone application (MetricWire, Inc., 2019) as part of an online study orientation. To ensure that all participants understood and followed the instructions, a brief comprehension quiz was administered after the orientation. Respondents were not allowed to participate until they passed the quiz.

Participants completed a 7-day^2^ event-contingent ambulatory assessment protocol and were asked to fill out a survey following any interpersonal interaction that lasted at least 5 min. In addition to situational attachment expectations, participants reported on perceptions of their interaction partner’s behavior and their own interpersonal behavior and affective experience during interactions (Kaurin, Beeney, Stepp et al., 2020; Kaurin, Dombrovski, Hallquist et al., 2020; Wright et al., 2017). Reports of *N*=3,971 interactions, based on *N*=263 participants, were collected with an average of 10.5 (*SD* = 7.27) interactions per participant. The average number of surveys completed across participants with a minimum of 10 interactions (*N*=3,574) was 19.53 (*SD* = 6.30).

#### State Attachment

We used nine items to assess momentary attachment expectations derived from the State Adult Attachment Measure (SAAM; Gillath et al., 2009). Based on face validity and the factor loadings and results reported in Gillath et al. (2009), we selected items that were most indicative of each attachment dimension and were likely to be responsive to interpersonal interactions. Reliability for included scales was demonstrated with McDonald’s omega values (*ω*; Zinbarg et al., 2005), calculated using the psych (Revelle, 2020) package in R. Each of the chosen SAAM items was re-worded to be reflective of feelings in a given interpersonal situation. For example, we re-phrased “I feel alone and yet don't feel like getting close to others” to “I felt alone and yet didn't feel like getting close to the other person.” Participants were asked to indicate how they felt during the interaction on a seven-point scale (0 =“disagree strongly” to 4=“agree strongly”). To be consistent with the scoring of the original scale, we created mean scores of items reflecting anxious (*ω*_within_=.86; *ω*_between_=.89), avoidant (*ω*_within_=.71; *ω*_between_=.90), and secure (*ω*_within_=.69; *ω*_between_=.92) attachment orientations. The full set of SAAM items used in this study can be found via https://osf.io/3vy4m/.

#### Affect

At each assessment, participants rated the degree to which they felt positive (i.e., happy, excited) and negative emotions (i.e., ashamed, nervous, hostile, sad, and angry) derived from the Positive and Negative Affect Schedule (Watson et al., 1988). Positively and negatively valenced items were separately averaged to create an overall negative or positive affect scale. Items read “How [ADJECTIVE] did you feel during the interaction?”, and ratings were made on a slider scale from 0 (“Not at All”) to 100 (“Extremely”) for each adjective (positive: *ω*_within_=.75; *ω*_between_=.91; negative: *ω*_within_=.71; *ω*_between_=.89).

#### Interpersonal Behavior

When participants indicated that an interpersonal interaction had occurred, they reported on their own behavior and the behavior of the person with whom they interacted. The item prompts were as follows: “Please rate how the OTHER PERSON BEHAVED toward you during the interaction” and “Please rate YOUR BEHAVIOR toward the other person during the interaction.” For both self and other, dominance was rated from “Accommodating/Submissive/Timid” to “Assertive/Dominant/Controlling” and warmth was rated from “Cold/Distant/Hostile” to “Warm/ Friendly/Caring” on 101-point slider scales (−50 to +50). These items were developed to reflect daily behavioral manifestations of affiliative and agentic motives described by interpersonal theory (Kiesler, 1996; Wiggins, 1991) and have demonstrated good construct validity in several samples (Woods et al., 2020).

#### Interaction Characteristics

To specify the relational context of their interactions, participants were also asked to describe some aspects of their interactions in detail. They were asked to indicate the interaction partner’s relationship to them, how frequently they interacted with the person, as well as to specify the kind of interaction they were having. In most instances, interaction partners were described as friends (47.18%), followed by a family member including parents (23.97%), significant others or casual romantic partners (10.27%), acquaintances (5.69%), or work- or school-related encounters (6.99%). The remainder were either persons that have not been met before (4.31%) or responses classified as “other” (1.5%). In general, interaction partners were persons with whom our participants were in touch on a daily basis (47.67%), or at least 2–6 days a week (29.45%). Approximately 11% reported being in touch with their interaction partners once a week and roughly ~11% 1–11 times a year. Most interactions took place in person (56.88%), 11.53% were phone calls, 15.03% text chats, 16.39% video calls, and 0.18% described as other. To statistically control for the salience of the interaction and relevance of the partners for one’s attachment system, we also asked participants to describe the closeness of the relationship within which the interaction was unfolding (“How close are you to the person you interacted with?”; 0—“Not at all close” to 100—“Extremely Close”). The mean value of this rating (*M*=67.43; *SD*=30.42) aligns with categorical data above, indicating that, on average, participants were interacting with close others.

### Data Analysis

To capture dynamic processes in the context of daily interpersonal interactions, we used multi-level structural equation models (MSEM; Sadikaj et al., 2021). Assessing attachment states across interpersonal interactions in daily life generates a hierarchical data structure, wherein social interactions are nested within individuals. MSEM allows the variability in individuals’ responses to be partitioned into between- and within-person components. Between-person variance reflects individual differences in average responses, and the corresponding portion of the model estimates associations among individual differences in each observed variable, akin to coefficients derived from cross-sectional designs. In contrast, within-person variance reflects interaction-to-interaction fluctuations around an individual’s average level, helping to determine whether specific relational events lead to contextual (as opposed to enduring) changes in working models.

This variance decomposition approach allows us to assess trait-like and contextual interpretations of working models of attachment, without putting them into opposition. That is, although it is likely that there is significant within-person variation in attachment patterns, that variation is best understood as a contextual deviation from a person’s typical attachment orientation (Sadikaj et al., 2021). Thus, the outcomes key to these analyses—i.e., the dynamic responses of one’s attachment system—are reflected in within-person results. These results allow us to articulate quantitatively how and to what extent attachment expectations fluctuate across daily interactions and how much a person’s own warm or dominant behavior covaries with such fluctuations. All within-person regression paths were estimated as random slopes, which allows us to test how much individuals differ in the strength and direction of their within-person associations. The fixed effects of these slopes represent the average association in the sample, and the random effects represent individual differences in the extent to which situational features influence participants.

Figure 1 provides a diagram of the focal model including an overview of momentary within-person links. We regressed ratings of momentary attachment experiences on perceptions of the other’s behavior during an interpersonal situation, a path we refer to as *attachment activation*. In our model, attachment expectations were situated as an intervening variable to account for the associations between perceptions of others’ and one’s own behavior. In line with CIIT, we refer to the path between perception of others and one’s own behavior as *interpersonal complementarity*. The link between attachment expectations and one’s own behavior is labeled *attachment manifestation*. At the between-person level, we estimated associations between average individual differences in perceptions of others, attachment expectations, and one’s own behavior. Because all variables were measured contemporaneously and it is assumed that they are mutually influential within interpersonal interactions, our study goal was not to model causality or the temporal ordering of the variables. Instead, the paths included in the models reflect this study’s emphasis on evaluating the unique effects of attachment on interpersonal behavior.

In subsequent sensitivity analyses, we examined further the specific effects of attachment expectations by parsing out variance attributable to other theoretically relevant constructs. To this end, we controlled for (negative and positive) affect (see grey paths in Figure 1), given the close relationship between attachment and affect-regulatory processes (Davila & Sargent, 2003). Because attachment-related dynamics may be influenced by longitudinal aspects of a relationship (Cook, 2000; La Guardia et al., 2000; Pierce & Lydon, 2001), we also accounted for the closeness of the relationship, at both levels of analysis. These sensitivity analyses are summarized in Tables 3 (negative affect), 4 (positive affect), and 5 (closeness).

Coefficients for covariates are not presented for parsimony. Along with other parameters not reported in the tables (e.g., residual variances), covariances among between-persons variables are not depicted in the diagrams, but full specifications and detailed output from all models can be found online at https://osf.io/3vy4m/. Table 1 summarizes pooled within-person correlations among the variables and correlations among the random intercepts at the between-person level.

**Table 1:**
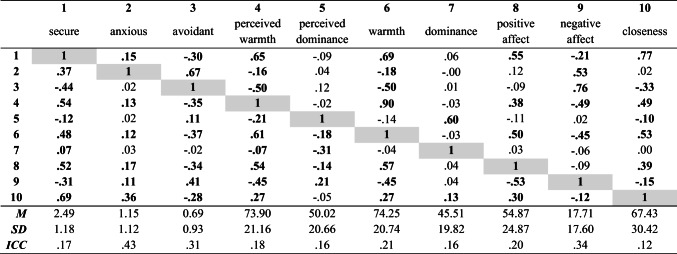
Correlations among study variables at within and between-person levels

*Note. N*_*between*_=263; *N*_*within*_=3971; values below diagonal represent within-person coefficients and values above diagonal represent between-person coefficients. Values in bold are those for which the credibility interval did not contain zero

All models were estimated in Mplus (version 8.4; Muthén & Muthén, 2019). Missing data were assumed to be missing at random. A Bayesian approach to SEM uses all available data in estimation which—with increasingly large samples—provides results comparable to those achieved with full information maximum likelihood to address missing data (Asparouhov & Muthén, 2010). Significance for all model parameters was based on 95% credibility intervals (CIs), with CIs that excluded zero indicating a parameter that differed significantly from zero. Sex (0=female; 1=male) and age (centered on mean age) were included as covariates in all models at the between-person level, and interaction number (i.e., daily number of interactions centered on mean number of observations per day) was entered as a within-person covariate.

## Results

Descriptive statistics and bivariate correlations are summarized in Table 1. Intraclass correlations (ICCs) are also presented in Table 1 indicating the proportion of between-person variance in each observed variable. Subtracting the ICC from 1.0 provides the within-person variance and indicates the proportion of state variance of a given construct. Overall, attachment expectations were characterized by differing levels of between-person and within-person variance. Descriptively, the lowest levels of stability emerged for secure attachment (ICC=.17), followed by avoidant (ICC=.31) and anxious (ICC=.43) attachment. To contextualize, ICCs for attachment were within the range of values established for negative (ICC=.34) and positive affect (ICC=.20) and interpersonal perceptions (ICC_*warmth*_=.18; ICC_*dominance*_=.16) and behaviors (ICC_*warmth*_=.21; ICC_*dominance*_=.16).

### Focal Model

In the models central to this paper, we tested whether the link between perceptions of others’ and one’s own behavior was accounted for by state attachment expectations. The results of this model are presented in Table 2. For models examining the effects of others’ perceived warmth on one’s own warm behavior, we found significant fixed effects at the within-person level (*interpersonal complementarity*), as well as the between-person level (Beh_i_ → Perc_i_). Perceiving others as more affiliative was associated with higher levels of attachment anxiety and security, and lower levels of avoidant attachment (*attachment activation* within-person path). The effect sizes for anxiety and security differed markedly, with the effect for security approximately 4 times that of anxiety. At the same time, avoidant attachment expectations were associated with less, while secure attachment expectations were associated with more interpersonally warm behaviors (*attachment manifestation *within-person path), over and above the effect of perceived warmth.

**Table 2:** Standardized key coefficients from focal multilevel models

	Affiliation	Dominance
	*β* [95% CIs]	*β* [95% CIs]
**Anxiety**		
Between-person		
Beh_i_ ↔ Perc_i_	**.90 [.85; .93]**	**.55 [.38; .67]**
Perc_i_ ↔ Attachment_i_	**−.18 [−.35; −.04]**	.03 [−.15; .20]
Attachment_i_ ↔ Beh_i_	**−.20 [−.35; −.07]**	−.01 [−.16; .16]
Within-person		
*interp. complementarity* (c)	**.61 [.58; .63]**	**−.28 [−.32; −.25]**
*attachment activation* (a)	**.13 [.09; .17]**	.00 [−.03; .04]
*attachment manifestation* (b)	.02 [−.02; .05]	.02 [−.02; .05]
**Avoidance**		
Between-person		
Beh_i_ ↔ Perc_i_	**.90 [.86; .93]**	**.58 [.42; .70]**
Perc_i_ ↔ Attachment_i_	**−.54 [−.65; −.41]**	.09 [−.09; .24]
Attachment_i_ ↔ Beh_i_	**−.52 [−.63; −.39]**	−.04 [−.19; .15]
Within-person		
*Interp. complementarity* (c)	**.56 [.53; .59]**	**−.28 [−.32; −.25]**
*Attachment activation* (a)	**−.28 [−.32; −.24]**	**.09 [.06; .13]**
*Attachment manifestation* (b)	**−.19 [−.23; −.06]**	−.03 [−.06; .01]
**Security**		
Between-person		
Beh_i_ ↔ Perc_i_	**.90 [.86; .94]**	**.55 [.39; .73]**
Perc_i_ ↔ Attachment_i_	**.66 [.57; .76]**	.12 [−.08; .29]
Attachment_i_ ↔ Beh_i_	**.67 [.58; .75]**	−.05 [−.24; .14]
Within-person		
*Interp. complementarity* (c)	**.52 [.48; .56]**	**−.28 [−.31; −.24]**
*Attachment activation* (a)	**.49 [.45; .52]**	**.07 [.04; .11]**
*attachment manifestation* (b)	**.19 [.07; .23]**	**−.06 [−.10; −.01]**

*Note. N*_*between*_=263; *N*_*within*_=3971; *Perc*, perceived affiliation or dominance; *Beh*, own affiliative or dominant behavior; *interp.*, interpersonal; values in bold are those for which the credibility interval did not contain zero

In a next step, perceived dominance and dominant behavior were examined as features of these same interpersonal processes. Perceiving interaction partners as more dominant was associated with less of one’s own dominant behavior at the within-person, but more dominant behavior at the between-person level. Thus, in the moment, when participants were perceiving someone as more dominant, they tended to behave in more submissive ways. However, those who generally perceived others as dominant, also tended to behave in more dominant ways, a general pattern indicative of anti-complementarity.

Together this suggests that while we adjust our agentic behavior towards complementarity in the moment, we may spend time with others who are more similar to us agentically. Perceptions of others as dominant were associated with higher levels of attachment security and avoidance (*attachment activation* within-person path). At the same time, secure attachment expectations were related to less interpersonally dominant behaviors (*attachment manifestation* within-person path), over and above the effect of perceived dominance; avoidant attachment expectations were not related to dominant behavior. Momentary activation patterns of anxious attachment, however, were neither related to perceptions of others as dominant, nor one’s own dominant behavior.

### Sensitivity Analysis

To check whether the observed effects were robust to the influence of alternative, theoretically plausible variables, we examined the extent to which they were affected by the simultaneous introduction of negative or positive affect or closeness. For this purpose, we included affect and self-reported closeness in separate models as parallel intermediate variables in the *interpersonal complementarity* path (see grey model parts in Fig. 1). The respective coefficients can be retrieved from Tables 3, 4 and 5.

**Table 3:** Standardized key coefficients from sensitivity analyses controlling for negative affect

	Affiliation	Dominance
	*β* [95% CIs]	*β* [95% CIs]
**Anxiety**		
Between-person		
Beh_i_ ↔ Perc_i_	**.87 [.79; .91]**	**.38 [.17; .55]**
Perc_i_ ↔ Attachment_i_	**−.27 [−.42; −.11]**	−.05 [−.23; .11]
Attachment_i_ ↔ Beh_i_	**−.24 [−.41; −.07]**	−.03 [−.19; .14]
NA_i_ ↔ Beh_i_	**−.50 [−.61; −.36]**	−.16 [−.32; .00]
Perc_i_ ↔ NA_i_	**−.46 [−.57; −.32]**	**−.25 [−.42; −.09]**
Attachment_i_ ↔ NA_i_	**.55 [.42; .66]**	**.51 [.39; .61]**
Within-person		
*Interp. complementarity* (c)	**.64 [.61; .66]**	**−.28 [−.31; −.25]**
*Attachment activation* (a)	**.16 [.11; .20]**	.03 [−.01; .07]
*Attachment manifestation* (b)	**.20 [.10; .24]**	−.01 [−.06; .03]
NA_it_ ➔ Beh_it_	**−.45 [−.48; −.42]**	**.20 [.16; .24]**
Perc_it_➔ NA_it_	**−.46 [−.50; −.43]**	**.04 [.00; .08]**
Attachment_it_ ↔ NA_it_	**.17 [.14; .21]**	**.11 [.08; .14]**
**Avoidance**		
Between-person		
Beh_i_ ↔ Perc_i_	**.87 [.82; .92]**	**.42 [.18; .59]**
Perc_i_ ↔ Attachment_i_	**−.52 [−.63; −.38]**	−.13 [−.29; .04]
Attachment_i_ ↔ Beh_i_	**−.50 [−.63; −.38]**	.01 [−.17; .18]
NA_i_ ↔ Beh_i_	**−.53 [−.64; −.39]**	**−.16 [−.32; .02]**
Perc_i_ ↔ NA_i_	**−.49 [−.61; −.36]**	**−.26 [−.42; −.09]**
Attachment_i_ ↔ NA_i_	**.75 [.67; .82]**	**.72 [.64; .79]**
Within-person		
*Interp. complementarity* (c)	**.64 [.61; .67]**	**−.28 [−.32; −.24]**
*Attachment activation* (a)	**−.37 [−.41; −.33]**	−.03 [−.07; .01]
*Attachment manifestation* (b)	**−.23 [−.27; −.09]**	.03 [−.02; .07]
NA_it_ ➔ Beh_it_	**−.35 [−.39; −.32]**	**.18 [.14; .22]**
Perc_it_➔ NA_it_	**−.48 [−.51; −.44]**	.04 [.00; .07]
Attachment_it_ ↔ NA_it_	**.28 [.25; .31]**	**.41 [.38; .43]**
**Security**		
Between-person		
Beh_i_ ↔ Perc_i_	**.85 [.76; .89]**	**.40 [.21; .59]**
Perc_i_ ↔ Attachment_i_	**.57 [.41; .68]**	.15 [−.05; .33]
Attachment_i_ ↔ Beh_i_	**.55 [.34; .65]**	.01 [−.18; .21]
NA_i_ ↔ Beh_i_	**−.52 [−.64; −.37]**	−.13 [−.31; .04]
Perc_i_ ↔ NA_i_	**−.46 [−.61; −.35]**	**−.27 [−.42; −.11]**
Attachment_i_ ↔ NA_i_	**−.21 [−.38; −.01]**	**−.28 [−.42; −.13]**
Within-person		
*Interp. complementarity* (c)	**.64 [.61; .67]**	**−.28 [−.32; −.25]**
*Attachment activation* (a)	**.51 [.47; .54]**	**.07 [.03; .11]**
*Attachment manifestation* (b)	**.43 [.14; .46]**	**−.04 [−.08; −.00]**
NA_it_ ➔ Beh_it_	**−.29 [−.34; −.26]**	**.18 [.14; .22]**
Perc_it_➔ NA_it_	**−.51 [−.54; −.44]**	.04 [−.00; .08]
Attachment_it_ ↔ NA_it_	**−.14 [−.17; −.10]**	**−.31 [−.34; −.28]**

*Note. N*_*between*_=263; *N*_*within*_=3971; *Perc*, perceived affiliation or dominance; *Beh*, own affiliative or dominant behavior; *NA*, negative affect; values in bold are those for which the credibility interval did not contain zero

**Table 4:** Standardized key coefficients from sensitivity analyses controlling for positive affect

	Affiliation	Dominance
	*β* [95% CIs]	*β* [95% CIs]
**Anxiety**		
Between-person		
Beh_i_ ↔ Perc_i_	**.87 [.79; .91]**	**.51 [.27; .67]**
Perc_i_ ↔ Attachment_i_	**−.26 [−.42; −.11]**	−.01 [−.18; .15]
Attachment_i_ ↔ Beh_i_	**−.23 [−.38; −.06]**	.00 [−.16; .17]
PA_i_ ↔ Beh_i_	**.22 [.02; .37]**	−.04 [−.20; .12]
Perc_i_ ↔ PA_i_	**.38 [.20; .51]**	.11 [−.06; .26]
Attachment_i_ ↔ PA_i_	.08 [−.11; .23]	.13 [−.03; .27]
Within-person		
*Interp. complementarity* (c)	**.63 [.60; .66]**	**−.29 [−.32; −.25]**
*Attachment activation* (a)	**.15 [.10; .20]**	.02 [−.02; .06]
*Attachment manifestation* (b)	**.04 [.01; .07]**	.03 [−.01; .07]
PA_it_ ➔ Beh_it_	**.54 [.51; .57]**	**−.13 [−.16; −.09]**
Perc_it_➔ PA_it_	**.60 [.57; .62]**	**.04 [.01; .08]**
Attachment_it_ ↔ PA_it_	**.13 [.10; .16]**	**.16 [.13; .19]**
**Avoidance**		
Between-person		
Beh_i_ ↔ Perc_i_	**.86 [.79; .91]**	**.52 [.29; .67]**
Perc_i_ ↔ Attachment_i_	**−.54 [−.66; −.40]**	−.09 [−.25; .10]
Attachment_i_ ↔ Beh_i_	**−.54 [−.67; −.39]**	.04 [−.12; .20]
PA_i_ ↔ Beh_i_	**.24 [.06; .42]**	−.04 [−.23; .15]
Perc_i_ ↔ PA_i_	**.40 [.21; .56]**	.09 [−.09; .26]
Attachment_i_ ↔ PA_i_	−.07 [−.26; .11]	−.11 [−.27; .07]
Within-person		
*Interp. complementarity* (c)	**.64 [.61; .66]**	**−.29 [−.32; −.25]**
*Attachment activation* (a)	**−.38 [−.42; −.33]**	−.04 [−.07; .01]
*Attachment manifestation* (b)	**−.21 [−.24; −.10]**	**.06 [.01; .10]**
PA_it_ ➔ Beh_it_	**.48 [.44; .51]**	−.10 [−.14; −.06]
Perc_it_➔ PA_it_	**.62 [.59; .64]**	.04 [.00; .07]
Attachment_it_ ↔ PA_it_	**−.16 [−.19; −.13]**	**−.35 [−.38; −.32]**
**Security**		
Between-person		
Beh_i_ ↔ Perc_i_	**.85 [.79; .90]**	**.49 [.28; .68]**
Perc_i_ ↔ Attachment_i_	**.58 [.45; .69]**	.15 [−.03; .31]
Attachment_i_ ↔ Beh_i_	**.54 [.40; .66]**	.02 [−.16; .19]
PA_i_ ↔ Beh_i_	**.25 [.05; .41]**	−.03 [−.21; .16]
Perc_i_ ↔ PA_i_	**.40 [.23; .54]**	.11 [−.06; .26]
Attachment_i_ ↔ PA_i_	**.47 [.30; .60]**	**.57 [.46; .68]**
Within-person		
*Interp. complementarity* (c)	**.64 [.61; .66]**	**−.28 [−.32; −.25]**
*Attachment activation* (a)	**.51 [.48; .54]**	**.07 [.03; .11]**
*Attachment manifestation* (b)	**.34 [.31; .38]**	**−.05 [−.09; −.00]**
PA_it_ ➔ Beh_it_	**.36 [.32; .40]**	**−.10 [−.14; −.06]**
Perc_it_➔ PA_it_	**.62 [.60; .65]**	**.05 [.01; .08]**
Attachment_it_ ↔ PA_it_	**.33 [.30; .36]**	**.51 [.48; .53]**

*Note. N*_*between*_=263; *N*_*within*_=3971; *Perc* perceived affiliation or dominance; *Beh* own affiliative or dominant behavior; *PA* positive affect; values in bold are those for which the credibility interval did not contain zero

**Table 5:** Standardized key coefficients from sensitivity analyses controlling for closeness of the relationship

	Affiliation	Dominance
	*β* [95% CIs]	*β* [95% CIs]
**Anxiety**		
Between-person		
Beh_i_ ↔ Perc_i_	**.88 [.82; .92]**	**.48 [.30; .63]**
Perc_i_ ↔ Attachment_i_	**−.26 [−.41; −.08]**	−.06 [−.22; .12]
Attachment_i_ ↔ Beh_i_	**−.23 [−.40; −.07]**	−.04 [−.20; .12]
Closeness_i_ ↔ Beh_i_	**.39 [.18; .53]**	−.03 [−.22; .14]
Perc_i_ ↔ Closeness_i_	**.43 [.22; .57]**	.08 [−.11; .26]
Attachment_i_ ↔ Closeness_i_	−.03 [−.24; .15]	.03 [−.16; .19]
Within-person		
*Interp. complementarity* (c)	**.63 [.60; .65]**	**−.29 [−.32; −.26]**
*Attachment activation* (a)	**.14 [.09; .18]**	.02 [−.02; .06]
*Attachment manifestation* (b)	**.06 [.02; .10]**	.04 [−.00; .08]
Closeness_it_ ➔ Beh_it_	**.25 [.21; .28]**	**−.06 [−.10; −.02]**
Perc_it_➔ Closeness_it_	**.31 [.27; .34]**	**.12 [.09; .16]**
Attachment_it_ ↔ Closeness_it_	**.33 [.30; .36]**	**.34 [.31; .37]**
**Avoidance**		
Between-person		
Beh_i_ ↔ Perc_i_	**.87 [.80; .92]**	**.46 [.28; .63]**
Perc_i_ ↔ Attachment_i_	**−.54 [−.65; −.39]**	−.12 [−.27; .02]
Attachment_i_ ↔ Beh_i_	**−.52 [−.65; −.38]**	.01 [−.16; .17]
Closeness_i_ ↔ Beh_i_	**.40 [.21; .56]**	−.05 [−.21; .14]
Perc_i_ ↔ Closeness_i_	**.43 [.24; .58]**	.09 [−.08; .29]
Attachment_i_ ↔ Closeness_i_	**−.41 [−.55; −.21]**	**−.35 [−.48; −.21]**
Within-person		
*Interp. complementarity* (c)	**.64 [.61; .66]**	**−.28 [−.32; −.25]**
*Attachment activation* (a)	**−.36 [−.39; −.31]**	−.04 [−.08; .01]
*Attachment manifestation* (b)	**−.32 [−.36; −.11]**	**.11 [.03; .16]**
Closeness_it_ ➔ Beh_it_	**.18 [.15; .24]**	−.02 [−.05; .02]
Perc_it_➔ Closeness_it_	**.33 [.29; .37]**	**.12 [.09; .16]**
Attachment_it_ ↔ Closeness_it_	**−.21 [−.24; −.18]**	**−.29 [−.32; −.26]**
**Security**		
Between-person		
Beh_i_ ↔ Perc_i_	**.86 [.80; .91]**	**.52 [.31; .67]**
Perc_i_ ↔ Attachment_i_	**.60 [.46; .70]**	.15 [−.03; .30]
Attachment_i_ ↔ Beh_i_	**.55 [.42; .67]**	−.01 [−.19; .17]
Closeness_i_ ↔ Beh_i_	**.39 [.23; .52]**	−.05 [−.23; .13]
Perc_i_ ↔ Closeness_i_	**.44 [.27; .56]**	.08 [−.11; .25]
Attachment_i_ ↔ Closeness_i_	**.72 [.61; .81]**	**.76 [.66; .83]**
Within-person		
*Interp. complementarity* (c)	**.64 [.59; .66]**	**−.28 [−.32; −.25]**
*Attachment activation* (a)	**.50 [.46; .53]**	**.07 [.03; .10]**
*Attachment manifestation* (b)	**.65 [.12; .69]**	**−.13 [−.18; −.05]**
Closeness_it_ ➔ Beh_it_	−.13 [−.16; .20]	.04 [−.02; .09]
Perc_it_➔ Closeness_it_	**.35 [.30; .39]**	**.12 [.08; .16]**
Attachment_it_ ↔ Closeness_it_	**.65 [.64; .67]**	**.68 [.66; .70]**

*Note. N*_*between*_=263; *N*_*within*_=3971; *Perc*, perceived affiliation or dominance; *Beh*, own affiliative or dominant behavior; values in bold are those for which the credibility interval did not contain zero

As evident, the overall pattern of results did not change. One exception was that the link between avoidant attachment states and perceived dominance disappeared when controlling for negative affect. At the same time, a small suppression effect emerged, in that a significantly positive link between avoidant attachment states and own dominance appeared when we controlled for positive affect and closeness of the relationship. Moreover, the anxious attachment manifestation path in interactions perceived as warm was larger when we controlled for affect. Positive and negative affect were generally associated in expected directions with attachment, such that momentary elevations of avoidant and anxious attachment were linked to more negative and less positive affect, whereas the reverse was true for momentary elevations of secure attachment. Two exceptions from this overall pattern were that at the between-person level no significant link between positive affect and anxious or avoidant attachment emerged. In line with previous work (Kaurin, Beeney, Stepp et al., 2020; Wright et al., 2017), negative and positive affect were associated in expected directions with affiliation and dominance.

The second robustness check examined the impact of closeness of the relationship within which the interaction was unfolding. Again, the pattern of results observed in Table 3 did not change, and closeness was related to attachment and interpersonal perceptions as well as behavior in theoretically plausible directions.

## Discussion

Attachment refers to the ways in which people manifest their need for relatedness and proximity to others, especially in times of emotional distress. In this study, we harnessed the potential of intensive longitudinal data to test the assertion that working models of attachment represent adaptive affect-regulatory systems, reflected in fluctuations across daily social interactions. Overall, we found that the situational activation of working models varied as a function of interpersonal perceptions, and that these patterns accounted, in part, for *interpersonal complementarity* in interactions that were perceived as warm, were accompanied by more positive and less negative affect, and when people were expressing warmth in response. Our findings demonstrate that situational attachment variability represents an adaptive process, which coordinates the regulation of interpersonal responses with implications for affective responding. This is important because our findings suggest that greater within-person fluctuation in situational attachment may not necessarily reflect inconsistent evaluations of others, which is often discussed in the context of personality pathology (e.g., Davila et al., 1997). Rather, distinctive experiences in close relationships activate different attachment expectations, thus setting adaptive processes of interpersonal responding *in motion*. The results help to characterize the interpersonal and affective context within which intra-individual variation in attachment expectations occurs. This perspective serves to balance the focus on how generalized patterns of attachment influence social behavior, which tends to inform much prior research (Smith et al., 2010; Kaurin, Beeney, Stepp et al., 2020).

More specifically, in this study, interactions characterized by warmth and positive affective experiences typically co-occurred with elevated levels of attachment security and lowered levels of attachment anxiety or avoidance, but only states of security and avoidance tended to co-occur with one’s own expressed warmth. The absence of a relationship between warmth and anxious attachment may imply that sometimes attachment anxiety is expressed in more warm behavior and sometimes in less.

Interactions characterized by interpersonal perceptions of dominance tended to co-occur with more avoidant, but not anxious, attachment states as well as more secure attachment. At the same time, secure attachment manifestations were linked to less of one’s own dominant behavior. Generally, this pattern aligns with previous work suggesting that the formation of stable romantic relationships is linked to enhanced attachment security and decreased attachment insecurity (Feeney & Noller, 1992; Hammond & Fletcher, 1991; Kirkpatrick & Hazan, 1994), and that attachment dynamics likely act as facilitators of therapeutic change (e.g., Bernecker et al., 2014). At the same time, the present results extend such lines of work by offering a methodology and analyses that illustrate change processes as an ensemble of directed activations of a person’s attachment system, that is, their interpersonal affect-regulatory system.

A key question arising from our analyses is whether attachment manifestations are a cause, in and of themselves, of interpersonal behaviors or whether they represent a correlate, but not a causal component of interpersonal perceptions and behaviors. Since all variables were measured contemporaneously, we can only conclude that attachment, affect, interpersonal perception, and behavior are reciprocally influential within an interaction. Such limitations remind us that although it is plausible that specific interpersonal interactions set the attachment system in motion, causal inferences, or assumptions about the temporal ordering of the variables should be made with caution. Instead, the paths included in the models reflect this study’s emphasis on evaluating the unique effects of attachment, the closeness of the relationship, as wells as affect on interpersonal behavior. Now that we have demonstrated that systematic variability within daily attachment states exists, specific situational contexts that may set one’s expectations for interpersonal affect-regulation in motion should be examined. Future work should also strive to better capture the timescale, functional relationships, and causal mechanisms of these processes, treat the attachment dimensions not as independent but synergistic and study potential interactions among them in predicting interpersonal behavior.

Because people tend to select themselves into situations that align with their usual patterns of thinking, feeling, and behaving, a high level of stability may be indicative of increased covariation among variables that shape an individual’s attachment expectations rather than less responsivity of the attachment system per se (Fraley & Brumbaugh, 2004). Higher levels of situational fluctuation in secure (70%) compared to anxious attachment (59%) may speak to this hypothesis. The high level of within-person variance may also be indicative of an individual’s exploratory drive—attachment security allows a person to explore the environment in unhindered fashion (Elliot & Reis, 2003). This pattern needs to be replicated in future studies, assessing how people gain novel experiential knowledge across situations or relationships within which change is likely to occur.

Two central implications for the robustness of our results emerge from a series of sensitivity analyses. First, because we were unable to directly compare attachment dynamics in interactions involving specific attachment figures (e.g., romantic partner, close friend), we addressed this limitation by statistically controlling for the closeness of the relationship with daily interaction partners. Because this analysis did not alter the pattern of our results, we conclude that the type of relationship did not substantially account for variance in attachment complementarity. Second, daily attachment processes independently contributed to interpersonal complementarity, over and above potential effects of positive and negative affect and the closeness of the relationship (*see also* Davila & Sargent, 2003). This result supports the assumption that attachment expectations reflect specific regulatory processes that complement general efforts at affect regulation.

Our findings underscore the importance of ecologically valid methods for naturalistic characterizations of responsive patterns of intra-individual variation in attachment expectations, and thus anticipations of affect-regulatory support. Our models were based on the perceptions of the individual as key predictors for two reasons. First, self-reports typically provide a more accurate assessment of processes that are low in observability (such as mental construal; Vazire, 2010). Second, interpersonal theory emphasizes the situation as construed by the individuals participating in it (Kiesler, 1996). However, our use of self-report also contributes to shared method variance between momentarily assessed variables. Attachment is often assessed by observation, and future work should use methods that capture more overt features of the situation and attachment manifestations. Perspectives that prioritize objective assessments of the situation (Rauthmann et al., 2014) may help disentangle true differences in the warmth and dominance of interpersonal interactions from perceived warmth and dominance as well as systematic differences in interaction partners that arise in the context of dispositionally insecure attachment. One possibility might be to use a yoked ambulatory assessment design, in which one person provides data about an event (e.g., conflict) and another is prompted to do the same (e.g., patient’s spouse).

We acknowledge that sampling may also have affected the generalizability of our results. Our data were drawn from a convenience sample (i.e., undergraduates from a large university), and on average, participants reported generally secure attachment experiences. Insecure attachment states, in contrast, were less frequently endorsed, which led to more skewed distributions. Moreover, a large proportion of our sample reported low numbers of interpersonal interactions (≤ 10), although the general pattern of our results was independent of the number of interactions per participant (*see footnote above*), and personality pathology (*see* *supplementary analyses*). Future studies should, therefore, focus on capturing systematic variation in the frequency of interactions in people’s daily lives, for example, by replicating our findings in clinical samples, where relationship experiences and affective reactivity play a significant role in the etiology and maintenance of disorders (e.g., Wright et al., 2017).

## Conclusion

Attachment expectations simultaneously arise from and influence the pattern and quality of social interactions in peoples’ daily lives. We provide evidence for reactive variation in attachment expectations across social interactions which, in turn, are associated with interpersonal complementarity. Our findings elaborate the conceptualization of working models of attachment as a mix of not only stable dispositions, but also contextual components related to the flow of daily experience. Building on CIIT, this work sheds light on attachment expectations as a key element linking perceptions of others with one’s own behavioral response. This approach allows us to identify dynamic elements of motivated interpersonal affect-regulation, with the hope that deeper a understanding of such processes will improve our clinical efforts to enhance interpersonal effectiveness and secure attachment.

## Supplementary Information

ESM 1 (DOCX 71 kb)

## Footnotes

1 For reasons of inter-study consistency and reliability, we usually exclude participants with low numbers of reported interactions (≤ 10) when estimating multilevel structural equation models. Because our analyses mainly focused on the within-person portion of the models, the results did not differ as a function of reported interactions per participant. Thus, at the request of an anonymous reviewer, we decided to deviate from this practice. Accordingly, results based on the subset with a minimum of 10 interactions per participant were moved to online supplementary materials at https://osf.io/3vy4m/ (i.e., syntax, output, APA formatted tables).

2 Although the protocol was designed for a period 7 days, the number of days in the study varied across participants. Some participants provided more than 7 days of assessments because the last data download for them did not transpire exactly on day 7 of the study.

## Additional Information

### Data Availability

All data have been made publicly available at the Open Science Framework (OSF) and can be accessed at https://osf.io/3vy4m/.
