# Supplementary material for: Attachment Manifestations in Daily Interpersonal Interactions
Source: Affect Sci. 2022 Jul 14;3(3):546–58. doi: 10.1007/s42761-022-00117-6 (PMC9537404; doi:10.1007/s42761-022-00117-6)
Supplement: Supplementary file 1 — (DOCX 71 kb) [file 42761_2022_117_MOESM1_ESM.docx]

**Supplementary Analysis Discussion**

In our data, the amount of reported social contact was unrelated to anxious (*r*=.02, *p*=.77) or avoidant (*r*=-.01, *p*=.87) attachment, nor did we find a statistically significant link to psychopathological dimensions relevant to the quality and frequency of interpersonal interactions such as negative affectivity (*r*=.06, *p*=.37), detachment (*r*=-.05, *p*=.46), antagonism (*r*=-.05, *p*=.39), disinhibition (*r*=-.12, *p*=.06) or psychoticism (*r*=-11, *p*=.07) as assessed by the Personality Inventory for DSM-5—Brief Form (PID-5; Krueger et al., 2012).

**Table S1**

Correlations among study variables at within and between-person levels.

|  | **1** | **2** | **3** | **4** | **5** | **6** | **7** | **8** | **9** | **10** |  |
| --- | --- | --- | --- | --- | --- | --- | --- | --- | --- | --- | --- |
|  | secure | anxious | avoidant | perceived warmth | perceived dominance | warmth | dominance | positive  affect | negative affect | closeness |  |
| **1** | **1** | **.17** | **-.26** | **.62** | -.09 | **.68** | .07 | **.58** | **-.18** | **.79** |  |
| **2** | **.36** | **1** | **.69** | **-.16** | .05 | -.15 | .02 | .11 | **.53** | .06 |  |
| **3** | **-.43** | .02 | **1** | **-.48** | .14 | **-.45** | .04 | -.11 | **.76** | **-.27** |  |
| **4** | **.54** | **.13** | **-.35** | **1** | -.02 | **.89** | -.05 | **.41** | **-.47** | **.49** |  |
| **5** | **-.12** | .03 | **.11** | **-.22** | **1** | **-.16** | **.66** | -.12 | .02 | -.15 |  |
| **6** | **.47** | **.11** | **-.36** | **.61** | **-.18** | **1** | -.04 | **.53** | **-.42** | **.54** |  |
| **7** | **.07** | .02 | -.01 | **-.08** | **-.29** | -.04 | **1** | .06 | -.04 | -.02 |  |
| **8** | **.53** | **.14** | **-.36** | **.55** | **-.16** | **.58** | .03 | **1** | -.09 | **.45** |  |
| **9** | **-.31** | **.12** | **.41** | **-.45** | **.22** | **-.44** | .04 | **-.59** | **1** | -.13 |  |
| **10** | **.69** | **.36** | **-.28** | **.27** | -.04 | **.27** | **.13** | **.29** | **-.11** | **1** |  |
| ***M*** | 2.47 | 1.14 | 0.69 | 73.76 | 50.09 | 74.15 | 45.42 | 54.43 | 17.91 | 67.54 |  |
| ***SD*** | 1.18 | 1.12 | 0.93 | 21.20 | 20.82 | 20.69 | 20.06 | 23.51 | 17.70 | 30.30 |  |
| ***ICC*** | .18 | .42 | .31 | .17 | .17 | .21 | .18 | .23 | .35 | .12 |  |

*Note.* *N_between_*=183; *N_within_*=3574; values below diagonal represent within-person coefficients and values above diagonal represent between-person coefficients. Values in bold are those for which the credibility interval did not contain zero.

**Table S2**

*Standardized Key Coefficients From Focal Multilevel Models.*

|  | **Affiliation** |  | **Dominance** |
| --- | --- | --- | --- |
|  | β [95% CIs] |  | β [95% CIs] |
| **anxiety** |  |  |  |
| **between-person** |  |  |  |
| Beh_i_ ↔ Perc_i_ | **.90 [.84; .94]** |  | **.61 [.44; .74]** |
| Perc_i_ ↔ Attachment_i_ | -.17 [-.35; .01] |  | .04 [-.14; .22] |
| Attachment_i_ ↔ Beh_i_ | -.16 [-.32; .02] |  | .01 [-.15; .17] |
| **within-person** |  |  |  |
| *interp. complementarity* (c) | **.60 [.57; .62]** |  | **-.26 [-.29; -.23]** |
| *attachment activation* (a) | **.13 [.09; .17]** |  | .02 [-.02; .05] |
| *attachment manifestation* (b) | .01 [-.02; .05] |  | .02 [-.01; .06] |
| **avoidance** |  |  |  |
| **between-person** |  |  |  |
| Beh_i_ ↔ Perc_i_ | **.89 [.83; .93]** |  | **.64 [.51; .78]** |
| Perc_i_ ↔ Attachment_i_ | **-.52 [-.65; -.38]** |  | .11 [-.09; .39] |
| Attachment_i_ ↔ Beh_i_ | **-.48 [-.62; -.34]** |  | .01 [-.16; .19] |
| **within-person** |  |  |  |
| *interp. complementarity* (c) | **.55 [.52; .58]** |  | **-.26 [-.29; -.22]** |
| *attachment activation* (a) | **-.28 [-.32; -.25]** |  | **.09 [.06; .13]** |
| *attachment manifestation* (b) | **-.18 [-.22; -.06]** |  | -.03 [-.07; .01] |
| **security** |  |  |  |
| **between-person** |  |  |  |
| Beh_i_ ↔ Perc_i_ | **.63 [.52; .75]** |  | **.61 [.44; .73]** |
| Perc_i_ ↔ Attachment_i_ | **.89 [.84; .93]** |  | .10 [-.08; .28] |
| Attachment_i_ ↔ Beh_i_ | **.67 [.57; .77]** |  | -.06 [-.27; .12] |
| **within-person** |  |  |  |
| *interp. complementarity* (c) | **.50 [.47; .56]** |  | **-.25 [-.29; -.22]** |
| *attachment activation* (a) | **.51 [.48; .54]** |  | **.08 [.04; .12]** |
| *attachment manifestation* (b) | **.19 [.06; .22]** |  | **-.06 [-.09; -.01]** |

*Note.* *N_between_*=183; *N_within_*=3574; Perc = perceived affiliation or dominance; Beh = own affiliative or dominant behavior; interp. = interpersonal; Values in bold are those for which the credibility interval did not contain zero.

**Table S3**

*Standardized Key Coefficients From Sensitivity Analyses controlling for Negative Affect.*

|  | **Affiliation** |  | **Dominance** |
| --- | --- | --- | --- |
|  | β [95% CIs] |  | β [95% CIs] |
| **anxiety** |  |  |  |
| **between-person** |  |  |  |
| Beh_i_ ↔ Perc_i_ | **.86 [.80;.91]** |  | **.50 [.31; .67]** |
| Perc_i_ ↔ Attachment_i_ | **-.24 [-.41;-.04]** |  | -.01 [-.21; .18] |
| Attachment_i_ ↔ Beh_i_ | **-.25 [-.41;-.05]** |  | -.01 [-.18; .18] |
| NA_i_ ↔ Beh_i_ | **-.51 [-.62;-.34]** |  | -.17 [-.34; .01] |
| Perc_i_ ↔ NA_i_ | **-.44 [-.59;-.28]** |  | **-.24 [-.41; -.05]** |
| Attachment_i_ ↔ NA_i_ | **.55 [.39;.65]** |  | **.50 [.33; .61]** |
| **within-person** |  |  |  |
| *interp. complementarity* (c) | **.62 [.60;.65]** |  | **-.26 [-.29; -.23]** |
| *attachment activation* (a) | **.14 [.10;.19]** |  | .03 [-.01; .06] |
| *attachment manifestation* (b) | **.20 [.08;.23]** |  | -.00 [-.05; .03] |
| NA_it_ 🡪 Beh_it_ | **-.44 [-.47;-.40]** |  | **.21 [.17; .25]** |
| Perc_it_🡪 NA_it_ | **-.44 [-.47;-.41]** |  | **.04 [.01; .07]** |
| Attachment_it_ ↔ NA_it_ | **.17 [.14; .20]** |  | **.11 [.08; .15]** |
| **avoidance** |  |  |  |
| **between-person** |  |  |  |
| Beh_i_ ↔ Perc_i_ | **.86 [.80;.91]** |  | **.55 [.32; .69]** |
| Perc_i_ ↔ Attachment_i_ | **-.45 [-.60;-.30]** |  | -.07 [-.24; .12] |
| Attachment_i_ ↔ Beh_i_ | **-.48 [-.63;-.34]** |  | .01 [-.18; .18] |
| NA_i_ ↔ Beh_i_ | **-.51 [-.65;-.36]** |  | **-.19 [-.38; -.01]** |
| Perc_i_ ↔ NA_i_ | **-.44 [-.58;-.29]** |  | **-.24 [-.39; -.07]** |
| Attachment_i_ ↔ NA_i_ | **.75 [.66;.83]** |  | **.72 [.63; .80]** |
| **within-person** |  |  |  |
| *interp. complementarity* (c) | **.62 [.60;.65]** |  | **-.25 [-.29; -.22]** |
| *attachment activation* (a) | **-.36 [-.39;-.32]** |  | -.02 [-.06; .02] |
| *attachment manifestation* (b) | **-.21 [-.25;-.07]** |  | .02 [-.03; .06] |
| NA_it_ 🡪 Beh_it_ | **-.35 [-.40;-.31]** |  | **.20 [.16; .24]** |
| Perc_it_🡪 NA_it_ | **-.45 [-.49;-.41]** |  | .04 [-.00; .07] |
| Attachment_it_ ↔ NA_it_ | **.29 [.25; .32]** |  | **.40 [.37; .43]** |
| **security** |  |  |  |
| **between-person** |  |  |  |
| Beh_i_ ↔ Perc_i_ | **.84 [.76;.90]** |  | **.51 [.33; .71]** |
| Perc_i_ ↔ Attachment_i_ | **.58 [.42;.70]** |  | .13 [-.08; .32] |
| Attachment_i_ ↔ Beh_i_ | **.54 [.35;.66]** |  | .01 [-.21; .22] |
| NA_i_ ↔ Beh_i_ | **-.49 [-.63;-.32]** |  | -.14 [-.33; .06] |
| Perc_i_ ↔ NA_i_ | **-.44 [-.58;-.30]** |  | **-.22 [-.38; -.06]** |
| Attachment_i_ ↔ NA_i_ | **-.19 [-.36;-.02]** |  | **-.24 [-.42; -.07]** |
| **within-person** |  |  |  |
| *interp. complementarity* (c) | **.63 [.60;.65]** |  | **-.26 [-.29; -.23]** |
| *attachment activation* (a) | **.50 [.46;.52]** |  | **.08 [.04; .12]** |
| *attachment manifestation* (b) | **.43 [.39;.46]** |  | -.04 [-.07; .00] |
| NA_it_ 🡪 Beh_it_ | **-.29 [-.33;-.26]** |  | **.19 [.14; .23]** |
| Perc_it_🡪 NA_it_ | **-.48 [-.52;-.44]** |  | .03 [-.00; .07] |
| Attachment_it_ ↔ NA_it_ | **-.14 [-.17; -.10]** |  | **-.31 [-.34; -.27]** |

*Note.* *N_between_*=183; *N_within_*=3574; Perc = perceived affiliation or dominance; Beh = own affiliative or dominant behavior; NA = negative affect; Values in bold are those for which the credibility interval did not contain zero.

**Table S4**

*Standardized Key Coefficients From Sensitivity Analyses controlling for Positive Affect.*

|  | **Affiliation** |  | **Dominance** |
| --- | --- | --- | --- |
|  | β [95% CIs] |  | β [95% CIs] |
| **anxiety** |  |  |  |
| **between-person** |  |  |  |
| Beh_i_ ↔ Perc_i_ | **.86 [.79;.91]** |  | **.57 [.38; .75]** |
| Perc_i_ ↔ Attachment_i_ | **-.22 [-.38;-.02]** |  | .01 [-.19; .19] |
| Attachment_i_ ↔ Beh_i_ | **-.24 [-.4;-.03]** |  | .03 [-.13; .23] |
| PA_i_ ↔ Beh_i_ | **.27 [.06;.45]** |  | -.05 [-.23; .15] |
| Perc_i_ ↔ PA_i_ | **.44 [.27;.60]** |  | .10 [-.08; .30] |
| Attachment_i_ ↔ PA_i_ | .11 [-.10;.30] |  | .17 [-.03; .33] |
| **within-person** |  |  |  |
| *interp. complementarity* (c) | **.62 [.59; .64]** |  | **-.26 [-.29; -.23]** |
| *attachment activation* (a) | **.14 [.10; .18]** |  | .02 [-.02; .06] |
| *attachment manifestation* (b) | **.05 [.01; .08]** |  | .03 [-.01; .07] |
| PA_it_ 🡪 Beh_it_ | **.53 [.50; .56]** |  | **-.14 [-.17; -.10]** |
| Perc_it_🡪 PA_it_ | **.58 [.56; .61]** |  | .04 [-.00; .08] |
| Attachment_it_ ↔ PA_it_ | **.13 [.09; .16]** |  | **.16 [.12; .19]** |
| **avoidance** |  |  |  |
| **between-person** |  |  |  |
| Beh_i_ ↔ Perc_i_ | **.85 [.77; .90]** |  | **.59 [.42; .73]** |
| Perc_i_ ↔ Attachment_i_ | **-.48 [-.63; -.30]** |  | -.05 [-.24; .13] |
| Attachment_i_ ↔ Beh_i_ | **-.52 [-.67; -.34]** |  | .05 [-.14; .21] |
| PA_i_ ↔ Beh_i_ | **.27 [.04; .45]** |  | -.06 [-.24; .18] |
| Perc_i_ ↔ PA_i_ | **.44 [.25; .58]** |  | .08 [-.11; .28] |
| Attachment_i_ ↔ PA_i_ | -.03 [-.22; .19] |  | -.07 [-.24; .11] |
| **within-person** |  |  |  |
| *interp. complementarity* (c) | **.62 [.60; .65]** |  | **-.26 [-.29; -.23]** |
| *attachment activation* (a) | **-.36 [-.40; -.32]** |  | -.03 [-.07; .01] |
| *attachment manifestation* (b) | **-.19 [-.23; -.15]** |  | **.05 [.00; .10]** |
| PA_it_ 🡪 Beh_it_ | **.46 [.43; .50]** |  | -.10 [-.14; -.07] |
| Perc_it_🡪 PA_it_ | **.60 [.57; .62]** |  | .03 [.00; .07] |
| Attachment_it_ ↔ PA_it_ | **-.17 [-.20; -.13]** |  | **-.35 [-.38; -.32]** |
| **security** |  |  |  |
| **between-person** |  |  |  |
| Beh_i_ ↔ Perc_i_ | **.85 [.77; .90]** |  | **.57 [.36; .71]** |
| Perc_i_ ↔ Attachment_i_ | **.59 [.44; .72]** |  | .12 [-.06; .34] |
| Attachment_i_ ↔ Beh_i_ | **.54 [.38; .68]** |  | .00 [-.23; .19] |
| PA_i_ ↔ Beh_i_ | **.28 [.06; .47]** |  | -.06 [-.22; .14] |
| Perc_i_ ↔ PA_i_ | **.45 [.25; .59]** |  | .09 [-.14; .27] |
| Attachment_i_ ↔ PA_i_ | **.49 [.27; .63]** |  | **.55 [.39; .68]** |
| **within-person** |  |  |  |
| *interp. complementarity* (c) | **.62 [.59; .64]** |  | **-.26 [-.29; -.23]** |
| *attachment activation* (a) | **.50 [.47; .53]** |  | **.08 [.05; .11]** |
| *attachment manifestation* (b) | **.35 [.27; .38]** |  | **-.04 [-.09; -.00]** |
| PA_it_ 🡪 Beh_it_ | **.35 [.32; .40]** |  | **-.11 [-.15; -.06]** |
| Perc_it_🡪 PA_it_ | **.61 [.58; .63]** |  | **.04 [.00; .07]** |
| Attachment_it_ ↔ PA_it_ | **.32 [.29; .35]** |  | **.50 [.48; .53]** |

*Note.* *N_between_*=183; *N_within_*=3574; Perc = perceived affiliation or dominance; Beh = own affiliative or dominant behavior; PA = positive affect; Values in bold are those for which the credibility interval did not contain zero.

**Table S5**

*Standardized Key Coefficients From Sensitivity Analyses controlling for Closeness of the Relationship.*

|  | **Affiliation** |  | **Dominance** |
| --- | --- | --- | --- |
|  | β [95% CIs] |  | β [95% CIs] |
| **anxiety** |  |  |  |
| **between-person** |  |  |  |
| Beh_i_ ↔ Perc_i_ | **.88 [.82; .92]** |  | **.58 [.43; .74]** |
| Perc_i_ ↔ Attachment_i_ | **-.21 [-.36; -.04]** |  | -.01 [-.21; .16] |
| Attachment_i_ ↔ Beh_i_ | **-.23 [-.38; -.03]** |  | .00 [-.18; .20] |
| Closeness_i_ ↔ Beh_i_ | **.44 [.26; .60]** |  | -.08 [-.29; .13] |
| Perc_i_ ↔ Closeness_i_ | **.46 [.30; .61]** |  | .04 [-.15; .27] |
| Attachment_i_ ↔ Closeness_i_ | .00 [-.18; .20] |  | .07 [-.13; .23] |
| **within-person** |  |  |  |
| *interp. complementarity* (c) | **.61 [.59; .64]** |  | **-.26 [-.29; -.23]** |
| *attachment activation* (a) | **.13 [.09; .17]** |  | .02 [-.02; .05] |
| *attachment manifestation* (b) | **.05 [.00; .09]** |  | .04 [-.00; .08] |
| Closeness_it_ 🡪 Beh_it_ | **.24 [.20; .28]** |  | **-.06 [-.09; -.02]** |
| Perc_it_🡪 Closeness_it_ | **.28 [.25; .32]** |  | **.12 [.09; .16]** |
| Attachment_it_ ↔ Closeness_it_ | **.33 [.30; .36]** |  | **.35 [.32; .38]** |
| **avoidance** |  |  |  |
| **between-person** |  |  |  |
| Beh_i_ ↔ Perc_i_ | **.87 [.79; .91]** |  | **.60 [.42**; **.75]** |
| Perc_i_ ↔ Attachment_i_ | **-.47 [-.60; -.32]** |  | -.08 [-.28; .10] |
| Attachment_i_ ↔ Beh_i_ | **-.51 [-.63; -.36]** |  | .02 [-.15; .19] |
| Closeness_i_ ↔ Beh_i_ | **.44 [.26; .61]** |  | -.07 [-.27; .16] |
| Perc_i_ ↔ Closeness_i_ | **.47 [.31; .64]** |  | .04 [-.14; .24] |
| Attachment_i_ ↔ Closeness_i_ | **-.34 [-.51; -.15]** |  | **-.31 [-.45**; **-.11]** |
| **within-person** |  |  |  |
| *interp. complementarity* (c) | **.62 [.59; .64]** |  | **-.26 [-.29; -.23]** |
| *attachment activation* (a) | **-.34 [-.38; -.30]** |  | -.03 [-.07; .01] |
| *attachment manifestation* (b) | **-.30 [-.34; -.06]** |  | **.11 [.03; .16]** |
| Closeness_it_ 🡪 Beh_it_ | **.17 [.14; .26]** |  | -.01 [-.05; .03] |
| Perc_it_🡪 Closeness_it_ | **.31 [.27; .35]** |  | **.12 [.09; .16]** |
| Attachment_it_ ↔ Closeness_it_ | **-.21 [-.24; -.17]** |  | **-.29 [-.32; -.26]** |
| **security** |  |  |  |
| **between-person** |  |  |  |
| Beh_i_ ↔ Perc_i_ | **.86 [.79; .90]** |  | **.61 [.41**; **.76]** |
| Perc_i_ ↔ Attachment_i_ | **.60 [.45; .72]** |  | .13 [-.08; .31] |
| Attachment_i_ ↔ Beh_i_ | **.55 [.40; .68]** |  | -.04 [-.23; .14] |
| Closeness_i_ ↔ Beh_i_ | **.44 [.28; .60]** |  | -.10 [-.29; .07] |
| Perc_i_ ↔ Closeness_i_ | **.47 [.32; .63]** |  | .04 [-.17; .24] |
| Attachment_i_ ↔ Closeness_i_ | **.77 [.67; .85]** |  | **.77 [.68; .85]** |
| **within-person** |  |  |  |
| *interp. complementarity* (c) | **.62 [.59; .64]** |  | **-.26 [-.29; -.23]** |
| *attachment activation* (a) | **.48 [.45; .51]** |  | **.08 [.04; .12]** |
| *attachment manifestation* (b) | **.64 [.56; .68]** |  | **-.13 [-.18; -.07]** |
| Closeness_it_ 🡪 Beh_it_ | **-.13 [-.18; -.08]** |  | **.04 [-.01; .10]** |
| Perc_it_🡪 Closeness_it_ | **.33 [.29; .37]** |  | **.12 [.08; .16]** |
| Attachment_it_ ↔ Closeness_it_ | **.65 [.63; .67]** |  | **.68 [.66; .70]** |

*Note.* *N_between_*=183; *N_within_*=3574; Perc = perceived affiliation or dominance; Beh = own affiliative or dominant behavior; Values in bold are those for which the credibility interval did not contain zero.
